# Spermatozoal Fractalkine Signaling Pathway Is Upregulated in Subclinical Varicocele Patients with Normal Seminogram and Low-Level Leucospermia

**DOI:** 10.1155/2017/5674237

**Published:** 2017-12-21

**Authors:** Salwa M. Abo El-khair, Mohammad A. Gaballah, Mamdouh M. Abdel-Gawad, Sherif Refaat M. Ismail, Ayman Z. Elsamanoudy

**Affiliations:** ^1^Department of Medical Biochemistry and Molecular Biology, Faculty of Medicine, Mansoura University, Mansoura, Egypt; ^2^Department of Dermatology, Andrology & STDs, Faculty of Medicine, Mansoura University, Mansoura, Egypt; ^3^Department of Dermatology and Andrology, Faculty of Medicine, Port Said University, Port Said, Egypt; ^4^Department of Clinical Biochemistry, Faculty of Medicine, King Abdulaziz University, Jeddah, Saudi Arabia

## Abstract

**Background:**

Fractalkine is produced in seminal plasma in small amounts and correlates with sperm motility.

**Purpose:**

To investigate the possible effect of low-level leucospermia on spermatozoa oxidative stress and sDNA fragmentation in patients with subclinical varicocele and apparently normal seminogram, and also to study the role of spermatozoal fractalkine and its receptor (CX3CR1) gene expression as a marker of spermatozoa inflammatory response.

**Methods:**

This study included 80 patients with subclinical varicocele (45 fertile and 35 infertile) and 45 age-matched fertile volunteers. In semen samples, fractalkine and CX3CR1 gene expression were investigated by qRT-PCR. Moreover, seminal plasma malondialdehyde (MDA) and total antioxidant capacity (TAC) were measured.

**Results:**

There are significant decrease in semen quality and significant increase in seminal leucocytes count in subclinical varicocele. Our results show a significant increase in MDA and TAC levels, DNA fragmentation, and expression levels of fractalkine and its receptor (CX3CR1) in subclinical varicocele groups.

**Conclusion:**

Subclinical varicocele induces seminal and spermatozoal subclinical inflammatory response in the form of low-level leucospermia and increased mRNA expression of the fractalkine signaling pathway, leading to increased spermatozoal ROS production, oxidative stress, and DNA fragmentation. These could cooperate in the pathogenesis of delayed fertility in males with subclinical varicocele.

## 1. Introduction

Subclinical varicocele is a condition in which varicose veins from the pampiniform plexus cannot be diagnosed by physical examination but need adjunctive diagnostic methods such as Doppler examination, color Doppler ultrasound, scrotal thermography, or venography [1]. Several studies have been conducted to explain the pathophysiology of testicular dysfunction occurring with varicocele. The exact mechanism of infertility caused by varicocele is not completely understood [2, 3].

Many hypotheses were postulated and investigated how varicocele could exert a harmful effect on spermatogenesis. These included semen oxidative stress state [4, 5], alterations in spermatozoa DNA integrity and mitochondrial activity [6]. Also, varicocele could decrease the testicular blood flow and renewal with a consequent accumulation of genotoxic substances [7].

Sperm DNA integrity is considered an indicator of normal spermatogenesis and fertility potential in males [8]. Damage of sperm DNA in patients with varicocele is correlated with levels of ROS production as well as varicocele degree [9]. Leucocytes (polymorphonuclear neutrophils and macrophages) have an important effect on male fertility as they are implicated in reactive oxygen species (ROS) production [10].

The WHO threshold for leucospermia was previously determined by 1.0 × 10^6^ WBC/mL or more [11]. Leucocyte count less than 1.0 × 10^6^ WBC/mL (low-level leucospermia) has a significant spermatozaoal damage effect in the form of decrease of motility and DNA integrity [12]. In addition, abnormality of sperm morphology at level of leucospermia as low as 0.5 × 10^6^ WBC/mL is reported [10]. Moreover, it is reported that low-level leucospermia is associated with increased seminal level of cytokines (IL-6 and IL-8) which could indicate and prove subclinical inflammation [13].

Fractalkine (CX3CL1) is the solitary member of the CX3C chemokine subfamily [14]. It exists in two forms: the membrane-anchored protein and the soluble form. The former is expressed in inflammatory endothelium and functions as an adhesion protein mediating the monocyte and T cell retention in inflamed tissue, while the soluble form is responsible for inducing chemotaxis. Chemotaxis and adhesion are mediated by the G protein-coupled receptor CX3CR1. Through both chemotactic and adhesive properties, CX3CL1 might have an important role in inflammation, and consequently, CX3CL1/CX3CR1 is involved in pathogenesis of various inflammatory disorders [15].

Fractalkine is produced in seminal plasma in small amounts and correlates with sperm motility [16]. Moreover, chemotaxis and thermotaxis of the sperm have been investigated previously in many studies [17, 18]. Zhang et al. [19] detected CX3CR1 mRNA and protein in spermatozoa, indicating that fractalkine may play a role in regulating sperm chemotaxis and maintaining its motility.

However, until now, to the best of our knowledge, no reported studies about spermatozoa fractalkine gene expression are published in spite of presence of data about its receptors.

So, in this work we aim at investigating the possible effect of low-level leucospermia on spermatozoa oxidative stress as well as sDNA fragmentation in patients with subclinical varicocele and apparently normal seminogram. Also, we aim at detecting the role of fractalkine and its receptors at the level of spermatozoal mRNA gene expression as a marker of spermatozoa inflammatory response in such patients.

## 2. Materials and Methods

### 2.1. Subjects Selection

The study is carried out on 125 participants: 80 individuals who were already diagnosed as patients with subclinical varicocele (45 fertile and 35 infertile) and 45 age-matched fertile volunteers with no clinical or sonographic signs of varicocele as a control group. All subjects gave written informed consent. All work was conducted in accordance with the Declaration of Helsinki (1964), and an approval was obtained from the Institutional Review Board (IRB) of Mansoura Faculty of Medicine.

The included infertile subjects have normal seminogram according to WHO [11]. They had attended the clinics of Andrology and Vascular Surgery Units, Mansoura University Hospital, from January 2014 to April 2015. They are married for more than one year with failed conception and unprotected regular intercourse. We excluded infertile couples that had female factors. An infertility sheet was obtained. Complete general and local genital examination was performed. Selected subjects had normal serum hormonal levels (FSH, LH, prolactin, T3, T4, TSH, estradiol and total and free testosterone). Scrotal color Doppler ultrasound was performed to confirm clinically detected varicocele and to diagnose subclinical one.

This research excluded any patient with semen abnormality, leucospermia (>1 × 10^6^/ml), or had infertility risk factor (gonadal toxins, cigarette smoking, use recreational drugs, alcohol intake, urogenital infection, clinically detected varicocele, undescended or small-sized testes, and cryptorchidism). Also, patients with any chronic disease (heart, kidney, or liver disease), endocrine disorder, acute or chronic inflammatory disease, or long-term medications (e.g., corticosteroids) were excluded.

Subclinical varicocele was diagnosed and graded by scrotal color Doppler ultrasonography according to classification of Sarteschi et al. [20]:

  Grade 1: venous reflux at the emergence of the scrotal vein only during the Valsalva maneuver; hypertrophy of the venous wall without stasis.
  Grade 2: supratesticular reflux only during the Valsalva maneuver; venous stasis without varicosities.
  Grade 3: peritesticular reflux during the Valsalva maneuver; overt varicocele with early-stage varices of the cremasteric vein.
  Grade 4: spontaneous basal reflux that increases during the Valsalva maneuver, possible testicular hypotrophy, overt varicocele, varicosities in the pampiniform plexus.
  Grade 5: spontaneous basal reflux that does not increase during the Valsalva maneuver, testicular hypotrophy, overt varicocele, varicosities in the pampiniform plexus.

The scrotal color Doppler ultrasonography maneuver was done according to the American Institute of Ultrasound in Medicine (AIUM) [21] and Italian Society for Vascular Investigation (SIDV-GIUV) [22].

### 2.2. Samples Collection

Semen samples were collected from the subjects attending the Infertility Clinic of Andrology Unit, Mansoura University Hospital. After sexual abstinence (3–5 days), semen samples were collected by masturbation.

### 2.3. Standard Semen Analysis

Seminal fluid was left for 1 hour at 37°C for liquefaction. Then, it was transferred to a test tube, and ejaculation volume was recorded. Sperms count and motility (total and progressive) were assessed with the motility/concentration module of the computer-assisted semen analysis (CASA) system using MiraLab–Egypt (Mira 9000 sperm Analyzer CASA software).

Morphology was investigated by smear preparation and sperm Mac stain method (Fertipro, Belgium) recommended by WHO [11]. Leucocytes count was identified by peroxidase staining technique as described by Politch and colleagues [23], which was firstly described by Endtz [24]. Viability was evaluated by Eosin Y staining with 100 cell score for stain uptake (dead cell) or exclusion (live cell) [25]. After semen sample liquefaction, seminal plasma was collected by centrifugation at 7000 rpm and stored as aliquots at −30°C until used for estimating 8-hydroxy-2′-deoxyguanosine (8-OHdG), malondialdehyde (MDA), and total antioxidant capacity (TAC).

### 2.4. RNA Extraction

One milliliter semen sample, after liquefaction, was added into tube with 2 ml RNAlater reagent (Sigma). Then, cells were pelleted by centrifugation. Following the manufacturer's instructions, total RNA was extracted from the sperm pellet using TriFast TM reagent (PeqLab. Biotechnologie GmbH, Carl-Thiersch Str. 2B 91052 Erlangen, Germany, Cat. No. 30-2010). Remaining DNAs were eliminated by digestion with DNase I (Sigma). Extracted RNA concentration and purity were determined by NanoDrop™ 2000 Spectrophotometer (Thermo Scientific, USA). Confirmation of the extracted RNA purity was done by formaldehyde agarose gel electrophoresis (2%) and ethidium bromide staining, to present 2 sharp bands (28S and 18S rRNA).

### 2.5. Fractalkine and CX3CR1 Genes Expression by Real-Time Quantitative RT-PCR

According to manufacturer's instructions, reverse transcription (RT) of the isolated RNA was carried out using Maxima First Strand cDNA Synthesis Kit for qRT-PCR (ThermoScientific, USA, cat No #K1641). The synthesized cDNA was stored at −20°C until use for qRT-PCR.

Primers for gene-specific qRT-PCR (purchased from Oligo™ Macrogen) were designed using the Primer3 software (v. 0.4.0) (http://frodo.wi.mit.edu/) to amplify human fractalkine (CX3CL1) with the following sequences: 5′-CTGCTGCCCTAACTCGAAAT-3′ (forward) and 5′-AGGACCACAGACTCGTCCAT-3′ (reverse) (PCR product: 103 bp), CX3CR1-specific primers were 5′-CACAAAGGAGCAGGCATGGAAG-3′ (forward) and 5′-CAGGTTCTCTGTAGACACAAGGC-3′ (reverse) (CX3CR1-product: 119 bp), while for *β*-actin, used as internal control (184 bp), 5′- AGAGCTACGAGCTGCCTGAC-3′ (forward) and 5′- AGCACTGTGTTGGCGTACAG-3′ (reverse).

The qRT-PCR reactions (25 *μ*L) were carried out, in duplicates, including 12.5 *μ*L Power Sybr® Green PCR Master Mix reaction buffer (Applied Biosystem), 10 pmol of forward and reverse gene-specific primers, and 2 *μ*L cDNA. The reaction cycling was 35 cycles (held for 15 sec at 95°C and for 30 sec at 60°C) after an initial one cycle at 95°C for 10 min. CT values (cycle threshold) were recorded. Melting curve analysis and 2% agarose gel electrophoresis were carried out to confirm PCR product specificity. No template negative control reaction was run in each experiment.

Relative quantification for fractalkine and CX3CR1 gene expression in semen samples was determined by the comparative ΔΔCT method. *β* actin was used as an internal control gene. For the overall change, calculation of ΔΔCT between cases and control samples was performed and linearized by 2^−ΔΔCT^ formula.

### 2.6. DNA Fragmentation Analysis

DNA fragmentation analysis was done by agarose gel electrophoresis [26]. Spermatozoa were collected after centrifugation, and DNA fragmentation was assessed by Enhanced Apoptotic DNA Ladder Detection kit (BioVision Research Products 980 Linda Vista Avenue, Mountain View, CA 94043, USA). In a 1.5 ml microcentrifuge tube, sperm pellet 5–10 × 10^5^ cells was washed with phosphate buffer saline, and the pellet was centrifuged for 5 min at 500 *g*. Supernatant was removed, and the cells were then lysed with 35 *μ*l Tris EDTA lysis buffer. 5 *μ*l of enzyme A reagent was added and incubated at 37°C for 10 min. Then, 5 *μ*l of enzyme B reagent was added and incubated at 50°C for 30 min. Ammonium acetate (5 *μ*l) and isopropanol (50 *μ*l) were added and mixed well. Washing of DNA pellet with 0.5 ml ethanol 70% was done and air-dried. Finally, dissolving DNA pellet in 20 *μ*l DNA suspension buffer was performed. The sample was loaded into a 1.8% agarose gel. The gel was stained by staining buffer (provided by the kit) with shaking gently for 30 minutes. DNA ladder was visualized with UV Transilluminator (Model TUV-20, OWI Scientific, Inc., 800 242-5560, France) and photographed.

### 2.7. Assay of Oxidative Stress Markers

Seminal plasma MDA [27] and TAC [28] were measured by colorimetric method using commercially available Kit (Cayman Chemical, Ann Arbor, MI, USA). Quantitative determination of seminal plasma 8-OHdG level was performed by Abnova 8-OHdG ELISA kit (Catalog number KA0444). The samples absorbance was determined using plate ELISA reader (Tecan, Sunrise Absorbance reader, Austria) at a 450 nm wave length.

### 2.8. Statistical Analysis

Data were tabulated, coded, and analyzed with the computer program SPSS (Statistical Package for Social Science) version 17.0. Descriptive statistics were presented as mean and standard deviation (mean ± SD). For statistical comparison, ANOVA (analysis of variance) test (for >2 groups of numerical parametric data) followed by post hoc was used. Pearson correlation coefficient test was used for different parameter correlation. The sensitivity and specificity were examined at different cutoff points using ROC curve analysis to determine the best cutoff point as well as the diagnostic power of each test. *P* value of  < 0.05 was considered statistically significant.

## 3. Results and Discussion

### 3.1. Results

The present study included 125 subjects with a mean age of 30.7 ± 11.3 years. The subjects were divided into 3 groups: 45 healthy subjects as controls and 80 patients with subclinical varicocele (45 fertile and 35 infertile). The subclinical varicocele patients were grade I or II by color Doppler ultrasound.

There are significant decrease in the quality of semen (concentration, normal morphology, motility, and vitality) and significant increase in the leucocytes count in the two subclinical varicocele groups in comparison to the control group. Also, there are significant decrease in the quality of semen and significant increase in the leucocytes count in the infertile group in comparison to the fertile group, but all parameters are still within normal values according to the normal reference ranges of WHO (2010) (Table 1).

**Figure 1:**
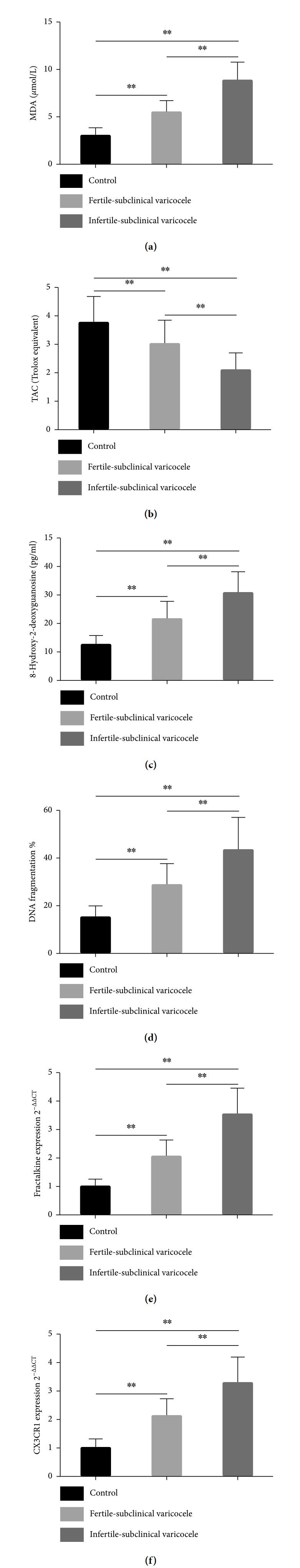
Oxidative stress state, DNA fragmentation, and fractalkine expression in subclinical varicocele patients. (a) Levels of MDA. (b) TAC. (c) 8-OHdG level. (d) DNA Fragmentation. (e) Fractalkine gene expression. (f) CX3CR1 gene expression. Data are represented in the form of mean ± SD. ^∗^ *p* < 0.05, ^∗∗^ *p* < 0.005, ^∗∗∗^ *p* < 0.0005.

Our results show that there are significant increases in MDA and 8-OHdG levels in subclinical varicocele groups in comparison to control and in the infertile group in comparison to the fertile group (Figures 1(a)–1(c)). Also, there is a significant decrease in TAC of subclinical varicocele groups in comparison to control and in the infertile group in comparison to the fertile group (Figure 1(b)).

There is a significant increase in the DNA fragmentation in subclinical varicocele groups in comparison to control and in the infertile group in comparison to the fertile group (Figure 1(d)). The expression levels of fractalkine and its receptor (CX3CR1) are significantly increased in subclinical varicocele groups in comparison to control and in the infertile group in comparison to the fertile group (Figures 1(e) and 1(f)).

**Figure 2:**
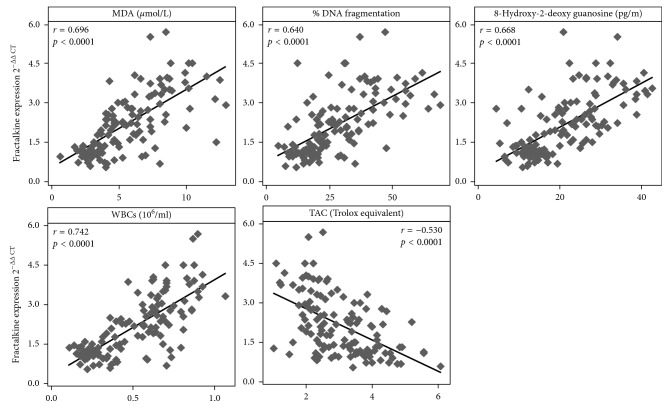
Correlation between fractalkine expression and other parameters. *r*: Pearson's correlation coefficient. *p*: Probability.

Moreover, results of the present study (Figure 2) show a strong positive correlation between fractalkine expression and MDA level, 8-OHdG level, DNA fragmentation, and seminal leucocytes counts. On the other hand, it shows a negative correlation with TAC.

**Figure 3:**
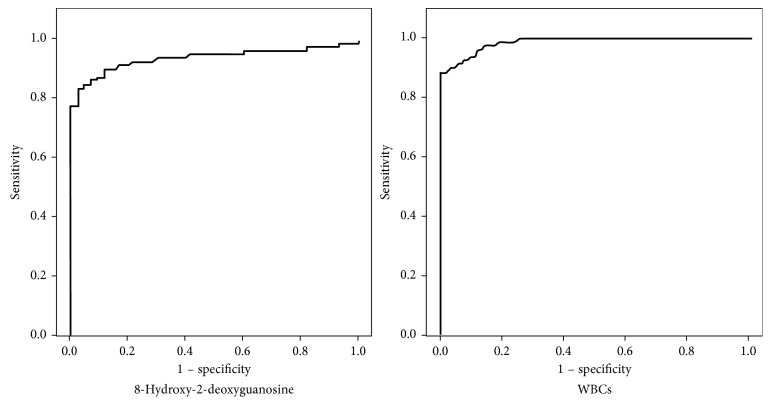
ROC curve analysis of 8-hydroxy-2-deoxyguanosine (8-OHdG) and seminal leucocytes count (WBCs).

The effectiveness of fractalkine expression and 8-OHdG in discriminating fertile from infertile men with different clinical diagnoses was studied by generating receiver operating characteristic (ROC) curves (Figure 3). The fractalkine expression level sensitivity was 92.5, and specificity was 91.1 (AUC = 95.0%, cutoff = 1.427) in discriminating controls from infertile patients. The 8-OHdG expression level sensitivity was only 87.5, and specificity was 91.1 (AUC = 93.8%, cutoff = 16.62) in discriminating controls from infertile patients. When setting the cutoff to 0.519, the seminal leucocytes count sensitivity was only 80.0, and specificity was 100.0 (AUC = 93.8%) (Table 2).

### 3.2. Discussion

Testicular dysfunctions that are associated with varicocele include elevated intratesticular temperature, developing testicular hypoxia, testicular gonadotoxins, and seminal of oxidants accumulation as well as evident production of anti-sperm antibodies. The documented pathophysiologic effects of varicocele could be suppressed activity of testicular DNA polymerase enzyme, induction of testicular apoptosis, and oxidative stress. Moreover, Sertoli and Leydig cell dysfunction and hormonal disorders were also reported [29].

The first objective of our study is to investigate the possible causative or associated relationship of low-level leucospermia and spermatozoa oxidative stress as well as sDNA fragmentation in patients with subclinical varicocele and apparently normal seminogram.

It is evident in the current study that subclinical varicocele is associated with spermatozoa oxidative stress which is presented by increased seminal plasma MDA and 8-OHdG with a significant increase in the percentage of sDNA fragmentation. On the other hand, there is a marked decrease in seminal TAC. The seminogram parameters also show a significant decrease in contrast to the controls in spite of the fact that it is still within normal level according to WHO [11] criteria. All of these findings are significantly more deteriorated in the infertile group of individuals when compared to the fertile peers.

The nonspecific seminal stress pattern in men with varicocele (either clinical or subclinical like our target group) is previously reported by Zümrütbaş et al. [30], documented by Pathak et al. [29] and confirmed in the current study by our mentioned findings. Moreover, spermatozoa oxidative stress is a dominant recognized molecular aberration in males with any degree of varicocele [31]. Spermatozoal oxidative stress could play an important role in pathogenesis of delayed fertility in such individuals [32].

Leucocytes and abnormal sperms are considered major sources of ROS in semen. Both are prominent features of varicocele [29, 31]. These coincide with our results.

Spermatozoa membrane and nuclear DNA damage caused by increased ROS with defective antioxidant defect could play a role in development of poor sperm quality including motility and fertilizing ability [33, 34]. Sperm mitochondrial and nuclear DNA are potential targets of attack by ROS [35] which usually progress to sperm apoptotic events that are completed in the epididymis during sperm maturation and capacitation [32].

Spermatozoa DNA fragmentation is associated with poor sperm function and quality regardless of the semen parameters. In most of the cases, seminogram shows a normal pattern on examination with CASA [36] as in our study but could be a main cause of delayed fertility in such individuals. So, the current study tested the specificity and sensitivity of 8-OHdG as a reliable sDNA damage marker in our target group. It shows 91.1% specificity and 87.5% sensitivity at cutoff level 16.62 pg/ml. It needs further investigation to confirm our result in larger number of subjects.

Also, we tested the specificity and sensitivity of low-level leucospermia as causative pathophysiologic mechanism in our target studied group (subjects with subclinical varicocele either fertile or infertile). ROC curve analysis revealed 100% specificity and 80.0% sensitivity at a cutoff level of 0.519 × 10^6^/ml. The result of the current study supports Agarwal et al. [10]. They reported a nearly similar result of leucospermia (0.5 × 10^6^/ml). Both results are much lower than that of WHO criteria of semen analysis [11] that documented 1.0 × 10^6^/ml is considered clinically significant and requires treatment. So, we could confirm the link between low levels of leucospermia and ROS generation [37] with its consequent pathological effects [13], especially sperm nuclear DNA fragmentation [38].

Like our study, Agarwal et al. [10] found no significant changes in semen parameters in individuals with low-level leucospermia from nonleucospermic subjects. Yet, ROS levels and the percentage of DNA damage were significantly high in the low-level leucospermia group. This supports that the level of leucospermia lower than the WHO criteria threshold [11] may have an impact on male fertility at the cellular and molecular levels rather than the seminogram parameters and may require treatment.

The concomitant presence of subclinical varicocele, low-level leucospermia, and sperm nuclear DNA fragmentation could play an important pathophysiologic mechanism of subfertility predisposition or even affect the male fertility potentials as presented in our study and documented previously by Agarwal et al. [10]. Alshahrani et al. [39] added another factor which is the advancing age. It was reported that all of these factors are associated with low fertilization rate, increased abortion risk, and incidence of diseases in offspring. They are also considered strong predictors of male fertility [39–41].

The five proposed and studied mechanisms of varicocele-induced delayed male fertility (hypoperfusion leading to hypoxia, heat stress, oxidative stress, hormonal imbalance, and exogenous toxins) still do not provide a full understanding. So, genetic and molecular factors might have a role in clarifying pathogenesis of varicocele-associated infertility [5, 42]. Consequently, the second objective of our work is to study the role of fractalkine and its receptors at the level of spermatozoal mRNA gene expression as a candidate molecular marker of spermatozoa inflammatory response.

The debate about the role of inflammation in varicocele pathogenesis of male subfertility took a long time of discussion. But it is documented that remarkable increase of ROS levels which can cause an inflammatory response detrimental to testicular tissue. It has been shown that varicocele increased ROS stress in a time-dependent manner. Varicocele-induced inflammation negatively impacted Sertoli cell physiologic function and may induce maturation arrest of spermiogenesis [43].

Many previous studies dealt with many seminal cytokines and inflammatory mediators in case of varicocele as nuclear factor-kappa B (NF-*κ*B) [44], interleukin-1*β* (IL-1*β*) [43], interleukin-6 (IL-6) and interferon-gamma [45], interleukin-37 (IL-37), interleukin-18-binding protein (IL-18BP), IL-18 receptor *β*, IL-18 [46], TNF-*α*, IL1*α*, IL6, Cd45, Cd3g, and Cd3d [47].

The novelty of our study is investigating spermatozoa fractalkine signaling pathway gene expression at the level of mRNA. There are no published data about this issue is documented.

Our results revealed increased spermatozoa mRNA expression of fractalkine and its coupled receptors (CX3CR1) in individuals with subclinical varicocele which is significantly higher in the infertile subgroup when compared to those of the fertile group. Their expression levels are positively correlated with MDA, 8-OHdG, WBCs count, and sperm nuclear DNA fragmentation % while it is negatively correlated with seminal TAC.

These results could prove their involvement in the pathophysiology of varicocele-induced spermatozoa subclinical inflammation and pathogenesis in male subfertility in such individuals.

The present study could conclude that subclinical varicocele induces seminal and spermatozoal subclinical inflammatory response in the form of low-level leucospermia and increased mRNA expression of the fractalkine signaling pathway. This inflammatory response leads to increased spermatozoal ROS production, oxidative stress, and nuclear DNA fragmentation. All of these interplay mechanisms could cooperate in the pathogenesis of delayed fertility in males with subclinical varicocele.

## Figures and Tables

**Table 1:** Semen parameters of all studied groups.

	Control	Fertile–subclinical varicocele	Infertile–subclinical varicocele
Number of cases	45	45	35
Volume (ml)	3.61 ± 0.73	4.26 ± 1.14^a^	5.02 ± 1.02^b,c^
Concentration (10^6^/ml)	117.11 ± 30.85	98.23 ± 17.09^a^	71.22 ± 19.04^b,c^
Normal morphology (%)	54.28 ± 18.29	37.09 ± 12.60^a^	16.49 ± 4.20^b,c^
% Motility (PR)	69.62 ± 13.91	50.50 ± 9.41^a^	33.58 ± 8.01^b,c^
Vitality (%)	56.51 ± 11.41	44.49 ± 10.11^a^	30.44 ± 8.07^b,c^
WBCs (10^6^/ml)	0.26 ± 0.08	0.57 ± 0.14^a^	0.75 ± 0.13^b,c^

Data are represented in the form of mean ± SD; ^a^significance between control group and fertile–subclinical varicocele group; ^b^significance between control group and infertile–subclinical varicocele group; ^c^significance between fertile–subclinical varicocele group and infertile–subclinical varicocele group; PR: progressive motility.

**Table 2:** ROC curve analysis of 8-OHdG and seminal leucocytes count.

	AUC (CI 95%)	Cutoff value	Sensitivity %	Specificity %	PPV %	NPV %	Accuracy %
8-OHdG	0.938 (0.89–0.98)	16.62	87.5	91.1	94.6	80.4	88.8
Seminal leucocytes count	0.988 (0.975–1.00)	0.519	80.0	100.0	100.0	73.8	87.2

AUC: area under the curve, CI: confidence interval, PPV: positive predictive value, NPV: negative predictive value.
